# Functional assessment of prevalent *kelch13* mutations reveals high-level artemisinin resistance potential in Bangladeshi *Plasmodium falciparum*

**DOI:** 10.1128/mbio.03691-25

**Published:** 2026-03-17

**Authors:** Maisha Khair Nima, Nirjhar Bhattacharyya, Douglas Shoue, Jungyoon Park, Ching Swe Phru, Saiful Arefeen Sazed, Prasad K. Padmanabhan, Sudhir Kumar, Mohammad Shafiul Alam, Michael Ferdig, Angana Mukherjee

**Affiliations:** 1 Department of Biological Sciences, University of Notre Dame 6111, Notre Dame, Indiana, USA; 2 Eck Institute of Global Health, University of Notre Dame 6111, Notre Dame, Indiana, USA; 3 Bloomberg School of Public Health, Johns Hopkins University 1466, Baltimore, Maryland, USA; 4 Infectious Diseases Division, International Centre for Diarrheal Diseases Research, Bangladesh (icddr,b) 56291 , Dhaka, Bangladesh; 5 Department of Biomedical Sciences, College of Veterinary Medicine, Iowa State University 1177, Ames, Iowa, USA; University of Geneva, Geneva, Switzerland

**Keywords:** artemisinin partial resistance, *kelch13* mutations, Chittagong Hill Tracts, Bangladesh, CRISPR-Cas9, ring-stage survival assay, fitness, drug resistance surveillance, malaria elimination

## Abstract

Artemisinin partial resistance (ART-R), driven by mutations in the *Plasmodium falciparum kelch13* (*k13*) gene, has contributed to malaria treatment failures in the Greater Mekong Subregion (GMS). The Chittagong Hill Tracts (CHTs) of Bangladesh account for nearly 90% of national malaria cases. Although K13-mediated ART-R has not been reported in the CHTs, its emergence remains a concern due to the region’s proximity to K13-mutation-endemic areas in Myanmar and India, and the presence of moderate K13-independent ART-R. A meta-analysis of global K13 mutation frequency identified C580Y, F446I, and R561H as the most prevalent substitutions, particularly in the GMS. To evaluate their potential impact, we introduced these mutations into two recent CHT isolates via CRISPR-Cas9: an artemisinin-sensitive strain and a moderately *in vitro* resistant, K13-independent strain (CHT-R). Edited lines were phenotyped using ring-stage survival assays (RSAs), post-treatment recovery, and competitive fitness assays. C580Y and R561H conferred marked increases in ART-R in both strains, while F446I had only a modest effect in CHT-R. All three mutations incurred minimal asexual fitness costs, except for F446I in the CHT-R background. R561H, the dominant allele at the Thai-Myanmar border and now expanding in Rwanda, conferred extreme resistance in CHT-R (RSA: 30.09% ± 1.9%), alongside an asexual growth advantage (fitness: 5.1% per generation) and the highest post-treatment recovery. This work provides the first functional evidence from South Asian parasites, including the first Asian analysis of K13 mutations in a resistant background, demonstrating that CHT parasites are genetically primed to sustain high-level ART-R, warranting enhanced surveillance during Bangladesh’s malaria elimination phase.

**IMPORTANCE:**

Artemisinin resistance threatens global malaria control and elimination efforts. While the Greater Mekong Subregion is already entrenched with kelch13 (K13)-mediated resistance, Bangladesh, though not yet reporting K13 mutations, has confirmed K13-independent artemisinin resistance in its endemic Chittagong Hill Tracts, which border resistance hotspots in Myanmar. This study is the first to functionally test globally dominant K13 mutations in Bangladeshi *Plasmodium falciparum* using genome editing. We show that mutations such as R561H and C580Y significantly increase resistance and asexual fitness, particularly in strains already carrying K13-independent resistance. These findings suggest that local parasites are genetically primed to acquire and sustain high-level resistance in asexual stages if these mutations emerge. Our work provides a timely warning and a framework for assessing drug resistance risk in other low-transmission regions with ongoing artemisinin use, critical for national elimination programs and global efforts to prevent the spread of resistance beyond Southeast Asia.

## INTRODUCTION

In 2015, World Health Organization member states adopted a strategy to reduce malaria incidence and mortality by at least 90% by 2030 (1). However, more than halfway to the target date, malaria remains a major global health threat, with 263 million cases and 597,000 deaths reported in 2023 (2). In the absence of an effective malaria vaccine, control and elimination efforts heavily rely on the clinical efficacy of artemisinin-based combination therapies (ACTs), which combine fast-acting artemisinin derivatives with partner drugs such as lumefantrine, amodiaquine, mefloquine, pyronaridine, sulfadoxine-pyrimethamine, or piperaquine (3).

In Bangladesh, where approximately 18 million people are at risk of malaria, substantial progress has been made in malaria control. From 2010 to 2020, the country achieved a 93% reduction in malaria cases and is actively working toward a complete national elimination by 2030 (4) by interrupting local transmission, preventing re-establishment, and impeding the emergence of ACT resistance. However, despite these efforts, *Plasmodium falciparum*, the primary cause of severe malaria and responsible for 80%–90% of malaria infections in Bangladesh (5–8), remains prevalent. Transmission is concentrated in the forested, mountainous Chittagong Hill Tracts (CHTs), comprising the districts of Bandarban, Rangamati, and Khagrachhari, which account for 80%–90% of the country’s malaria cases (5, 9, 10).

The spread of antimalarial drug resistance is a critical barrier to malaria elimination globally (11). In the late 2000s, partial artemisinin resistance (ART-R) emerged in Cambodia, characterized by prolonged parasite clearance times in patients, following treatment with artemisinin derivatives or ACTs (12–15). Genome-wide association studies subsequently linked delayed clearance to mutations in the *pfkelch13* (*k13*) propeller domain (16–18). Although ART-R can have a multigenic basis (19–25), K13 substitutions are the major genetic determinant of resistance, originally identified through *in vitro* evolution and genome sequencing, confirmed through field studies (26) and subsequently verified through reverse genetics (27) and experimental genetics (28). The standard *in vitro* correlate for ART-R, the ring-stage survival assay (RSA), measures early ring-stage-specific resistance by exposing parasites to pharmacologically relevant dosage of active metabolite; dihydroartemisinin (DHA), during the first 6 h of the asexual cycle (29). To date, over 200 propeller domain K13 substitutions have been found globally, of which only 14 have been validated as contributing to ART-R as measured by *in vitro* and *ex vivo* RSA per WHO guidelines (27, 30–33). These substitutions include P441L, F446I, C469Y, N458Y, M476I, Y493H, R539T, I543T, P553L, R561H, P574L, C580Y, R622I, and A675V.

In the Greater Mekong Subregion (GMS), the emergence and spread of ART-R-associated K13 mutations, coupled with resistance to partner drugs, have led to ACT failure rates up to 50% (34). These mutations have disseminated across eastern GMS countries via clonal expansion (Cambodia, Vietnam, Laos, and Thailand) (19, 35–37) and have independently emerged in Myanmar, China-Myanmar border, and Thai-Myanmar border (37–39). K13 substitutions have also been reported in northeastern India bordering the CHTs (40, 41). This pattern resembles historical resistance trends, notably chloroquine and sulfadoxine-pyrimethamine resistance, that spread from Southeast Asia through Bangladesh and India to Africa, causing significant morbidity and mortality (42). More recently, independent K13 mutations with clinical and *in vitro* evidence of ART-R have also emerged in South America, East Africa, and the Horn of Africa, further broadening global concern (30, 43–54).

Despite no current detection of K13-mediated ART-R (55–57), the CHTs in southeastern Bangladesh remain highly vulnerable due to their geographic proximity to K13-associated ART-R-prevalent regions in Myanmar and India and shared climatic conditions, seasonal transmission patterns, and malaria control challenges (58). Other malaria-endemic countries with similar timeline of ACT usage (37, 39, 54, 59–62) and transmission settings (9, 63–65) have also witnessed expansion and spread of K13-associated ART-R. This collective evidence underscores the high likelihood that the CHTs could experience the emergence and expansion of ART-R through both regional spread and independent evolution, posing significant threats to Bangladesh’s malaria elimination efforts.

In 2018 and 2019, we assessed ACT susceptibility in malaria patients with *P. falciparum* mono-infections from Bandarban and Alikadam districts of the CHTs using clinical parasite clearance assays. The median time to clear half of the initial parasite load was 5.6 h (range: 1.5–9.6 h), with 20% of patients exhibiting a median of 8 h (55). Additionally, we also detected quantifiable *in vitro* resistance by RSA that was independent of K13 substitutions.

Given these findings, the main objective of the current study was to proactively assess the phenotypic and fitness consequences of three highly prevalent K13 substitutions (F446I, R561H, and C580Y) using genome editing in two CHT patient isolates collected in 2018 and 2019 (55). These mutations were selected for their high prevalence, their historical emergence in both proximal and distal locations, and their temporal expansion. Prior to editing, we genetically and phenotypically characterized the isolates and identified one artemisinin-sensitive strain (CHT-S) and one K13-independent, *in-vitro* moderately resistant strain (CHT-R). We then introduced each mutation into both backgrounds and systematically assessed the impact of each mutation and the influence of background on ART-R and asexual growth using quantitative RSAs, post-treatment recovery assays, and competitive fitness assays. We demonstrate that K13 substitutions incur no to minimal fitness costs in both genetic backgrounds, with the exception of F446I in the CHT-R background, which shows a growth disadvantage. Strikingly, R561H mediates extreme resistance and growth advantage in the CHT-R background.

## RESULTS

### Spatio-temporal analysis of the prevalence of WHO-validated K13 substitutions in malaria-endemic regions

**Fig 1:**
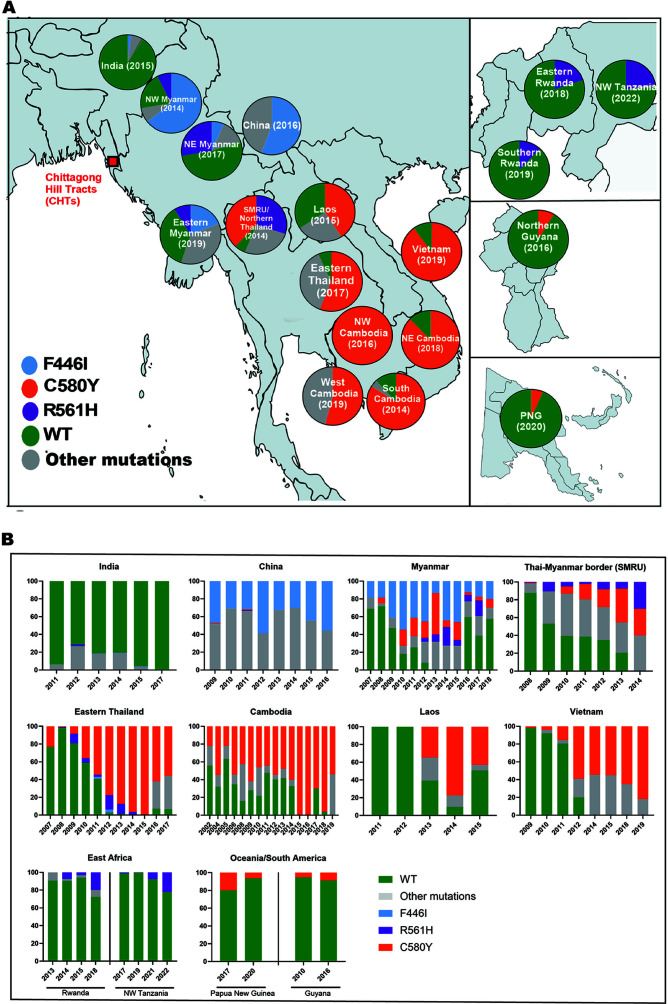
Frequency of commonly emerged and expanded K13 substitutions observed globally. We utilized WWARN Artemisinin Surveyor as the foundation, since it robustly summarizes the prevalence of *k13* molecular markers in the propeller region of the *k13* gene, extracted from published studies. These results were supplemented with additional information from publications not covered in WWARN (see Materials and Methods). (**A**) Colored pie charts in sectioned maps of the globe showing the prevalence of K13 substitutions: F446I, R561H, and C580Y in the GMS, R561H in Rwanda and Tanzania, C580Y in Guyana and Papua New Guinea, with the sampling year in parentheses for each region. Sampling years vary by country, reflecting the most recent data available as of October 2023. Total samples (*n*): India, *n* = 147; NW Myanmar, *n* = 210; NE Myanmar, *n* = 122; eastern Myanmar, *n* = 142; SMRU (northern Thailand), *n* = 30; Laos, *n* = 1,394; Thailand, *n* = 9; NW Cambodia, *n* = 3; west Cambodia, *n* = 74; south Cambodia, *n* = 76; NE Cambodia, *n* = 48; Vietnam, *n* = 50; eastern Rwanda, *n* = 73; southern Rwanda, *n* = 66; NE Tanzania, *n* = 176; northern Guyana, *n* = 144; PNG, *n* = 94; and China, *n* = 34. (**B**) Temporal prevalence of F446I, R561H, and C580Y in key regions of emergence. See Files S1 and S2 for the raw data. Total samples (*n*) per region and per year for the temporal analysis are included in File S2.

Fourteen K13 substitutions, validated and documented by WHO as markers of ART-R, exhibit varying prevalence across malaria-endemic regions worldwide. We conducted a comprehensive analysis of their frequency using data from the Worldwide Antimalarial Resistance Network (66), supplemented by recent studies (43, 47, 51, 52, 67–70) (Source Table S1). We analyzed 72,375 samples collected from regions where WHO-validated K13 substitutions have independently emerged or spread, spanning data from 1997 to the most recent sampling year for each country, covering 7 countries in Southeast Asia and South Asia, 14 countries in Africa, Papua New Guinea in Oceania, and Guyana in South America. The *k13* 3D7 reference sequence was present in the majority of samples (88.7%, *n* = 72,375) (Table S1). Cumulative prevalence of each of the K13 propeller domain substitutions (Table S1) demonstrated that C580Y (49.88%), F446I (14.40%), and R561H (7.65%), followed by P441L (6.59%), R539T (4.29%), and Y493H (3.09%), were the most frequent mutations. Next, we narrowed our focus to a detailed temporal and spatial analysis of these 14 WHO-validated mutations over the last 15 years (2007–2022), a period chosen because this was the inflection point where the total number of K13 mutations started increasing (Fig. S1; Source Table S1). To provide a global snapshot of these three most prevalent K13 substitutions—C580Y, R561H, and F446I, we analyzed their frequency across the GMS and regions of independent emergence, including East Africa, Guyana, and Papua New Guinea. Figure 1A presents the most recent K13 prevalence data available for each region at the time of data access (October 2023), and therefore, the sampling years differ across countries depending on when surveillance was last conducted, while Fig. 1B illustrates temporal trends.

C580Y has been the predominant K13 substitution in the GMS for the past decade, reaching full fixation (100%) in northwest (NW) Cambodia by 2016, 84.23% in south Cambodia by 2014, 87.5% in northeast (NE) Cambodia by 2018, and 54.05% in west Cambodia by 2019 (Fig. 1A; File S1). In Thailand, Laos, and Vietnam, C580Y remained prevalent at 40%–80% (2015–2017) (Fig. 1A; File S1). Figure 1B shows that C580Y outcompeted other mutations and WT alleles by 2014 in Thailand and Cambodia. Independent emergences recorded in Guyana around 2010 and Papua New Guinea around 2017 have maintained low but stable prevalence over the next years (Fig. 1).

F446I is dominant in Myanmar, particularly near the China-Myanmar border, reaching 64% in 2014 in NW Myanmar (Fig. 1A) and 19% in eastern Myanmar in 2019 (Fig. 1A). It has also been detected in Northeast India near the Bangladesh border (Fig. 1A). F446I was present in 20% of the Myanmar population by 2007 and increased to 54.5% by 2010; however, unlike C580Y, it did not outcompete other mutations or wild-type alleles (Fig. 1B; File S2). Its dominance along the Myanmar-China border and presence in Northeast India highlight its regional impact.

R561H remains prevalent at ~33% near the Thai-Myanmar border (SMRU/Northern Thailand). It is also high in Myanmar, especially in NE Myanmar (27.7%), ranking as the second most common mutation in Myanmar (Fig. 1A; File S1). In Thailand, the small proportion of the R561H mutation was replaced by C580Y between 2009 and 2015 (Fig. 1B; File S2). Outside the GMS, R561H has independently emerged as the most prevalent mutation in Rwanda, with a significant frequency increase observed in 2018. In NW Tanzania, which borders Rwanda, R561H has also emerged and expanded since 2018 (Fig. 1B; File S2).

### Genomic and *in vitro* phenotypic characterization of an artemisinin-sensitive and an *in vitro* K13-independent moderate artemisinin-resistant strain

The CHTs border northeastern India and Myanmar, where K13-mediated ART-R is prevalent (40, 62, 71). Approximately 90% of Bangladesh’s *P. falciparum* malaria cases occur within the forested, hilly CHTS, comprising three districts, including our specific study site in Bandarban. ART-R-associated K13 substitutions have not yet been reported from the CHTs or elsewhere in Bangladesh (55–57). In this study, we re-analyzed 1,310 samples from the broader Chittagong Division, covering the three CHT districts (Bandarban, Rangamati, and Khagrachari), as well as eight additional surrounding districts (Table S2). These samples were collected between 2008 and 2018 and are part of the MalariaGEN Pf7 data set (72). We identified five samples harboring the K13 substitution A578S and seven additional K13 substitutions: T573I, S711T, P475S, N523D, G674R, E605K, and A724V, each detected in only one sample (Table S2). Previous K13 surveillance studies from Bangladesh reported only A578S and did not identify any validated K13 markers of ART-R (55–57). None of these substitutions detected from the MalariaGEN analysis are associated with or validated for ART-R, and their lack of expansion suggests that they are not driven by ART selection pressure in the population. Although K13 resistance-associated mutations remain unreported in Bangladesh (55–57), our previous study in Bandarban (2018–2019) identified K13-independent ART-R in an isolate (55). We short-term lab-adapted 22 *P. falciparum* isolates collected from patients with mono-infections in Bandarban during 2018–2019, as previously described (55), for *in vitro* genetic and phenotypic profiling. From this cohort, we selected two isolates, I-001 and I-029, for in-depth genotypic and phenotypic drug resistance analysis (Tables 1 and 2; Table S3). Microsatellite genotyping revealed that I-029 was polygenomic; we derived a clonal population by limiting dilution and confirmed clonality by microsatellite analysis (73). To test ART-R, we used RSA where 0–h ring-stage parasites are subjected to 700 nM DHA treatment for 6 h, and survival rates are calculated 72 h later. A threshold of >1% survival is considered resistant (29). I-001, hereafter referred to as CHT-S, was artemisinin-sensitive with an RSA of 0.6% ± 0.13% (55) (Table 1), while I-029, referred to as CHT-R, exhibited moderate *in vitro* ART-R with a survival rate of 6.4% ± 0.53% (Table 1). However, both CHT-S and CHT-R had parasite clearance half-lives (PC_t1/2_) < 3 h (Table 1) (55), below the 5.5 h threshold cutoff. However, PC_50_ (the time for 50% parasite clearance) better captured differences between isolates under these conditions. The PC_50_ was markedly prolonged in CHT-R (7.68 h) compared with CHT-S (0.015 h) (Table 1) (55).

**TABLE 1:** Phenotypic characterization of CHT-S and CHT-R *Plasmodium falciparum* isolates from the CHTs used in this study^*a*^

Characteristic	Drug	CHT-S (I-001)	CHT-R (I-029)
Collection year	NA^*f*^	2018	2019
Collection site	NA	Bandarban Sadar Hospital	Bandarban Sadar Hospital
Parasite clearance half-life (PCt1/2, h)^*b*^	Artemether + lumefantrine	3.3	2.39
50% parasite clearance time (PC50, h)^*b*^	Artemether + lumefantrine	0.015	7.68
*In vitro* RSA (% survival)	Dihydroartemisinin	0.66 ± 0.13^*b*^	6.4 ± 0.53
*In vitro* IC50 (nM)^*c*^	Artemisinin	2 ± 2	2.5 ± 1.6
	Chloroquine	544 ± 54.29	386.5 ± 27.23
	Piperaquine	4.45 ± 0.74	3.49 ± 1.26
	Cycloguanil	7,832.88 ± 1.2	362.69 ± 49.7^*d*^
	Lumefantrine	1.02 ± 0.23	0.39 ± 0.14
	Mefloquine	31.51 ± 8.25	16.67 ± 2.55
	Amodiaquine	5.63 ± 0.23	2.39 ± 0.67^*e*^

^*a*^ The table summarizes the strain characteristics, clinical drug phenotypes, and *in vitro* drug phenotypes of isolates CHT-S (I-001) and CHT-R (I-029).

^*b*^ Also in reference 55.

^*c*^ Mean ± SEM of IC50 of each drug was calculated from three to five biological replicates conducted in duplicates. A Welch’s unpaired *t*-test was performed for analyzing significance.

^*d*^ *P *= 0.0034.

^*e*^ *P *= 0.0064.

^*f*^ "NA," not applicable.

**TABLE 2:** Genotypic characterization of drug-resistant markers from CHT-S and CHT-R isolates^*a*^

Gene name	Gene ID	CHT-S (I-001)	CHT-R (I-029)
Kelch 13 (K13)	PF3D7_1343700	WT^*b*^	WT
crt (chloroquine resistance transporter)	PF3D7_0709000	CVMNT (72–76), Q271E, R371I	CVMNT (72–76), Q271E, R371I
mdr1 (multidrug resistance protein 1)	PF3D7_0523000	WT	N86Y
AAT1 (amino acid transporter 1, putative)	PF3D7_0629500	F313S	S258L
dhfr (dihydrofolate reductase)	PF3D7_0417200	N51I, C59R, S108N, I164L	N51I, C59R, S108N
dhps (dihydropteroate synthase)	PF3D7_0810800	A581G	S436A, K540E
mdr2 (multidrug resistance protein 2)	PF3D7_1447900	F423Y, G299D, S208N	F423Y, G299D, S208N
cytb (cytochrome b)	mal_mito_3	WT	WT
carl (cyclic amine resistance locus)	PF3D7_1113300	K903E	K903E

^*a*^ Genotypes of known drug resistance markers, associated with artemisinin and other antimalarial drug resistance from whole-genome sequencing.

^*b*^ "WT” indicates absence of nonsynonymous mutations relative to the 3D7 reference sequence.

Whole-genome sequencing of both clones confirmed a wild-type *k13* sequence (3D7 reference), which was further validated by Sanger sequencing (Table 2). In 72 h *in vitro* drug testing, both isolates were sensitive to artemisinin, resistant to chloroquine and cycloguanil (the active form of proguanil) (Table 2), and carried key resistance-associated mutations, including *pfcrt* K76T/Q271E/R371I and a quadruple mutant *pfdhfr* haplotype (IRNL) (Table 2). CHT-S retained a wild-type *pfmdr1* (3D7-like), whereas CHT-R carried the N86Y mutation (Table 2). Additional genotypes in other candidate ART-R genes are listed in Table S3.

**Fig 2:**
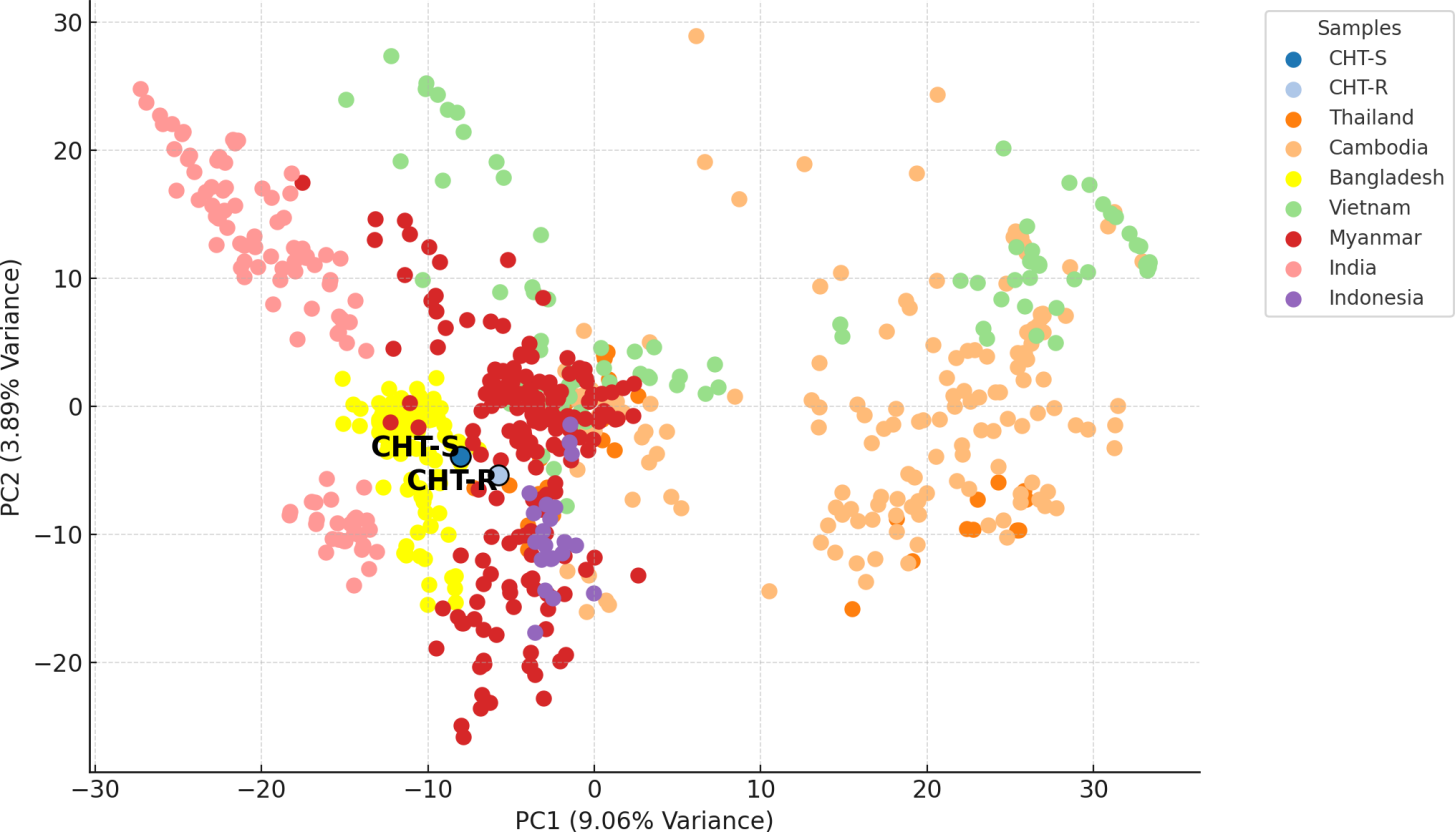
Principal component analysis of our whole-genome-sequenced CHT samples merged with other available *P. falciparum* genomes from Bangladesh and neighboring countries. PCA of the CHT-S and CHT-R and publicly available genomic sequences from the MalariaGEN Pf7 data set, from Chittagong (a division covering the CHTs and the coastal plains) in Bangladesh and neighboring Myanmar, India, Thailand, Cambodia, Vietnam, and Indonesia, with representative samples from each country, collected from 2008 to 2018, including a total of 696 samples. The first two principal components (PC1 and PC2) explain 9.06% and 3.89% of the variance of the multisample genomic data.

To investigate the population structure and genetic relationships among CHT-S, CHT-R, and neighboring regions, we conducted principal component analysis (PCA) (Fig. 2). We compared CHT-S and CHT-R with publicly available genomic sequences from the MalariaGEN Pf7 data set, from the Chittagong Division in Bangladesh, as well as neighboring Myanmar, India, Thailand, Cambodia, Vietnam, and Indonesia, with representative samples from each country, collected from 2008 to 2018, including a total of 696 samples. The CHT-S and CHT-R genomes are distinct from each other and cluster closely with 93 other Bangladeshi and 198 Myanmar isolates. Metadata of all the samples from MalariaGEN Pf7 used for PCA are present in Source Table S2. The samples from Myanmar display a broader distribution in the PCA space, indicating greater genetic diversity relative to Bangladesh. The additional pairwise PCA plots (PC1 vs PC3 and PC2 vs PC3) in Fig. S2A further illustrate these patterns, showing consistent clustering of the CHT samples with other Bangladeshi/Myanmar isolates across three principal component axes, which supports the robustness of this genetic grouping. Figure S2B shows the variance explained by the first 10 principal components, out of which the first three explain most of the variance.

### C580Y and R561H drive higher resistance in the CHT-S and CHT-R strains, while F446I shows a minimal effect

**Fig 3:**
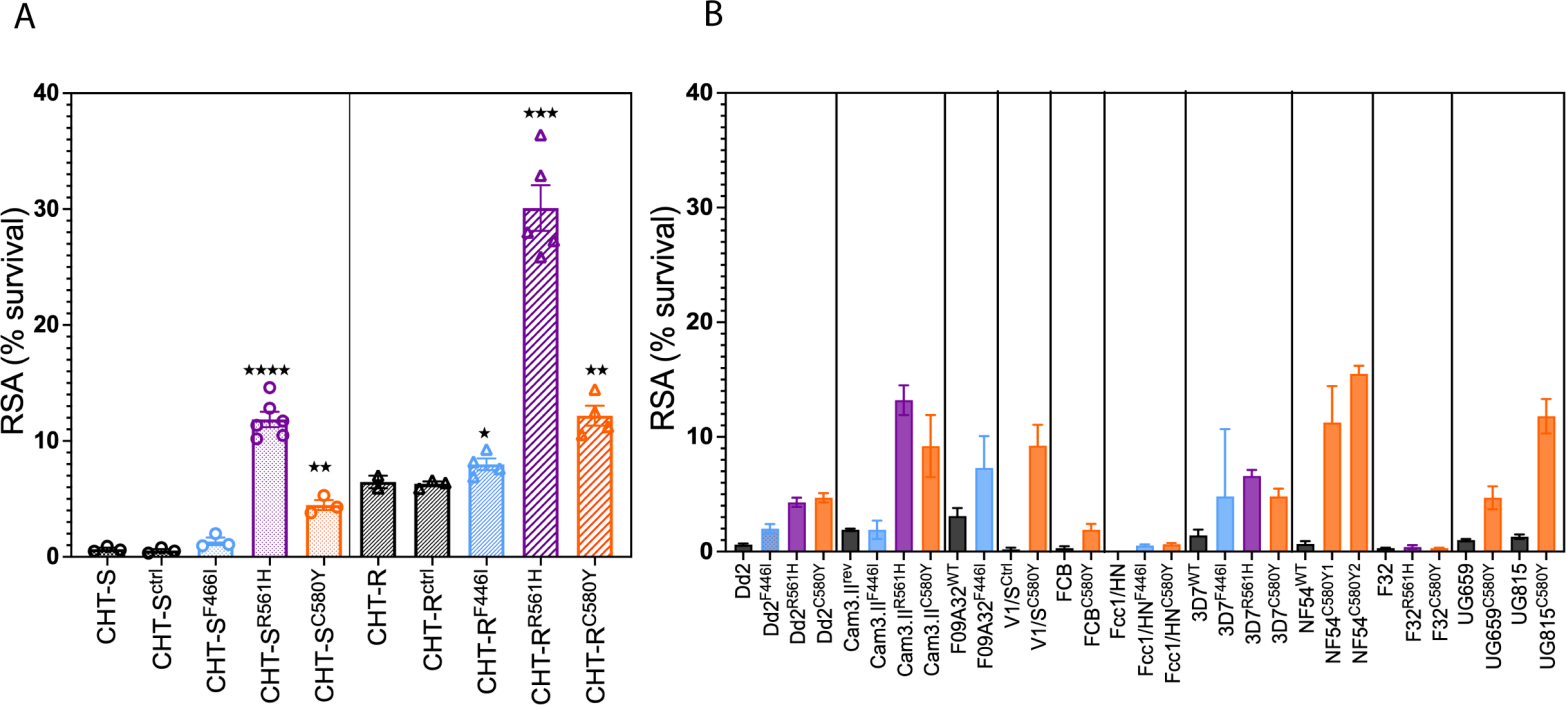
*In vitro* ART susceptibility (by RSAs) in genome-edited K13 F446I, R561H, and C580Y mutations in various genetic backgrounds. (**A**) RSA survival of F446I, R561H, C580Y, and control silent edits in the CHT-S (left panel) and in the CHT-R (right panel) backgrounds. Results show the percentage of early ring-stage parasites (0–3 h post-invasion) that survived a 6 h pulse of 700 nM DHA, relative to dimethyl sulfoxide-treated parasites assayed in parallel. Percent survival values are shown as means ± SEM, with results obtained from three to six independent experiments, each performed in duplicate. *P* values were determined by unpaired *t*-tests (with Welch’s correction) using CHT-S^ctrl^ and CHT-R^ctrl^ (harboring *k13* silent shield mutations at the *k13* gRNA cut site) as the comparators for all edits in the respective backgrounds. *P < 0.05; **P < 0.01; ***P < 0.001; and ****P < 0.0001. (**B**) Meta-analysis of the RSAs from the same *k13* genetic edits across different backgrounds, including laboratory strains and field-adapted strains from Cambodia, Tanzania, Uganda, China-Myanmar border, and reference strains from Vietnam, Hainan province of China, Indo-China, and West Africa (27, 30–32, 74, 75). See File S3 and File S4 for the values of the RSA data.

To isolate the causative effects of three globally prevalent nonsynonymous K13 substitutions (F446I, R561H, and C580Y) on ART-R in the CHT context, we used CRISPR-Cas9 to introduce each mutation into the CHT-S and CHT-R isolates. As editing controls, we also introduced two synonymous shield mutations in *k13* that do not alter amino acid sequences. Successful edits were confirmed by Sanger sequencing (Fig. S3), and independent clones for each edited line were obtained via limiting dilution and used for further analysis. Before assessing the impact of these K13 substitutions in the CHT-edited lines, we validated our RSA by testing previously published Dd2 lines carrying R561H and C580Y mutations, whose RSA phenotypes were consistent with prior reports (31) (Fig. S4). The edited CHT lines were then subjected to RSA, i.e., 700 nM DHA treatment during the 0–3 h ring stage, and percent survival was quantified 72 h later. Figure 3A and File S3 show the survival rates of *k13*-edited CHT strains. Control lines (CHT-S^ctrl^: 0.54% ± 0.14% [mean ± SEM] RSA survival; CHT-R^ctrl^: 6.30% ± 0.21% [mean ± SEM]), which carry only synonymous *k13* mutations, exhibited no significant difference in survival compared to their respective parent lines (CHT-S: 0.66% ± 0.13%; CHT-R: 6.47% ± 0.54%; unpaired *t*-test, ns) and were used as comparators in all statistical analyses. Figure 3B and File S4 present a meta-analysis of RSA survival rates of these *k13* edits across different genetic backgrounds. C580Y, the most prevalent and independently emerged substitution, produced RSA survival rates of 4.47% ± 0.4% (mean ± SEM) in CHT-S, representing an eightfold increase in mean resistance compared to control line CHT-S^ctrl^ (unpaired *t*-test, *P* < 0.001, Fig. 3A) and comparable to the resistance potential of C580Y in Dd2 (Fig. S4), as well as 3D7 and Ugandan backgrounds (UG659^C580Y^ and Dd2^C580Y^) in other studies (Fig. 3B, orange bars; File S4). In CHT-R, C580Y showed RSA levels of 12.18% ± 0.9% (mean ± SEM), indicating a twofold increase in resistance compared to CHT-R^ctrl^ (unpaired *t*-test, *P* < 0.001), similar to resistance levels observed in other backgrounds such as NF54 and Cam.3.II (Fig. 3B). Notably, R561H exhibited significantly high survival rates in both CHT-S and CHT-R backgrounds, with a 22-fold increase in resistance (11.86% ± 0.7%, unpaired *t*-test, *P* < 0.0001) in CHT-S and a 4.8-fold increase (30.09% ± 1.97%, unpaired *t*-test, *P* < 0.0001) in CHT-R compared to their respective control lines (Fig. 3A). The CHT-R background exhibited the highest RSA survival following introduction of the R561H mutation, surpassing all previously reported *k13*-edited lines (Fig. 3B, purple bars; File S4). To our knowledge, this represents an unprecedented resistance level for any genetically engineered *k13* line to date. F446I had a minimal impact on elevating survival in both CHT-S and CHT-R backgrounds (Fig. 3A). In CHT-S, F446I demonstrated an RSA of 1.34% ± 0.33% (unpaired *t*-test, ns) (Fig. 3A), similar to the resistance level observed in F446I-engineered Dd2 and Cam3.II backgrounds (Fig. 3B, blue bars). In CHT-R, F446I had a modest but significant effect on elevating survival, with a rate of 7.99% ± 0.5% (unpaired *t*-test, *P* = 0.03) compared to its isogenic edited control (Fig. 3A).

### K13 substitutions confer minimal fitness costs in Bangladeshi CHT *P. falciparum* isolates

To assess the asexual fitness impact of K13 substitutions, we performed replicated head-to-head growth competition assays. Tightly synchronized isogenic clones, differing by only one *k13* allele (*k13*-edited control and the other being a missense *k13*-edited variant), were co-cultured at a 1:1 ratio and maintained for 40 days. The relative growth of each clone was determined by quantifying the proportion of the corresponding *k13* allele using qPCR with a RhAmp SNP kit (IDT) (see Materials and Methods).

**Fig 4:**
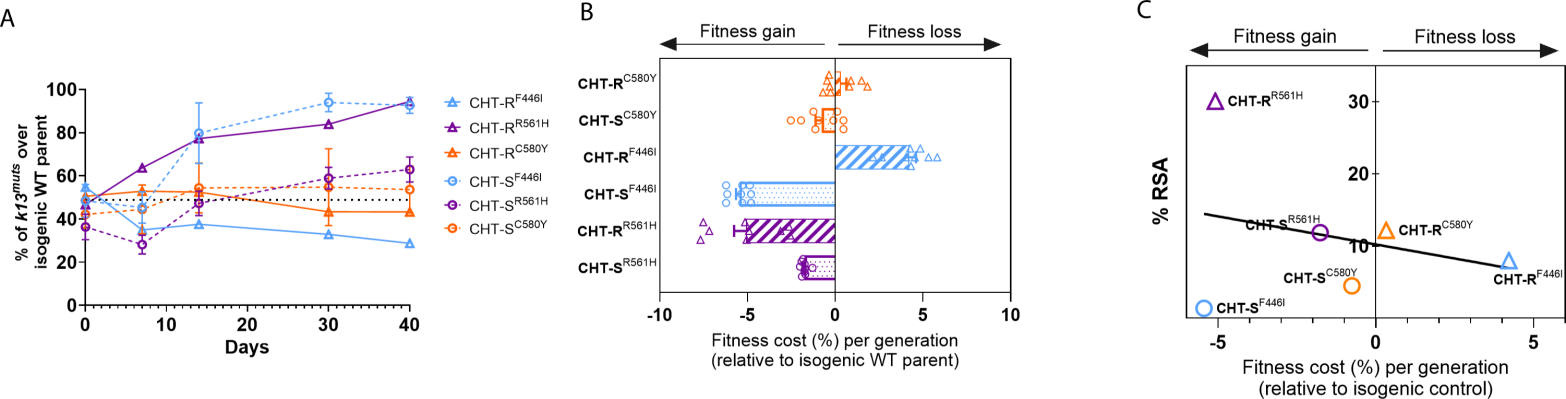
Head-to-head competitive fitness assays in the edited lines show no or minimal fitness costs. (**A**) Percentages of mutant *k13* alleles relative to the silent-edited control in the respective background over time. CHT-S^ctrl^ and CHT-R^ctrl^ were mixed at a 1:1 proportion with each of their edited lines (F446I/C580Y/R561H) on day 0 and cultured in 96-well plates for 40 days. Results shown as means ± SEM were obtained from three biological replicates performed in technical triplicate. Values are provided in File S5**.** (**B**) Percent difference in growth rate per 48 h generation, expressed as fitness costs, for CHT-S and CHT-R mutant lines relative to their isogenic silent-edited control lines, expressed as mean ± SEM. (**C**) Fitness costs of the mutant lines plotted against their corresponding RSA survival values.

The competitive growth assay showed distinct competition trajectories for F446I, R561H, and C580Y in both the CHT-S and CHT-R backgrounds, reflecting mutation- and background-specific differences in how each edited line competed against its isogenic control (Fig. 4A; File S5). Statistical significance was evaluated using the non-parametric Wilcoxon signed-rank test. In the CHT-S background, F446I provides an asexual growth advantage, reaching ~94% allele percentage by day 30 (*P* = 0.008). The proportion of R561H and C580Y remained relatively stable over time, with only slight fluctuations (Fig. 4A; File S5). In contrast, in the CHT-R background, the proportion of F446I declined 29% by day 30 (*P* = 0.0039). C580Y showed minimal fluctuations over the 40 days of competition, while R561H provided an asexual growth advantage, reaching 94% allele percentage by day 40 (*P* = 0.0039) (Fig. 4A; File S5).

From these data, we calculated the percent increase in growth rate per 48 h generation, expressed as fitness costs, for each *k13* mutant line relative to its isogenic control (Fig. 4B), where a negative fitness cost value signifies a fitness advantage. Fitness gain/cost per generation was minimal in these edited lines and varied from −5.44% to + 4.22%. F446I in CHT-S demonstrated a minimal fitness advantage (5.44% ± 0.2% per generation), and although it showed minimal fitness cost per generation in CHT-R (4.22% ± 0.4%), the cumulative effect resulted in a growth disadvantage during competition. C580Y demonstrated fitness neutrality in both CHT-S (−0.76% ± 0.3% per generation) and CHT-R (0.33% ± 0.3% per generation). R561H demonstrated a minimal fitness advantage in CHT-R (5.09% ± 0.7% per generation) but was fitness neutral in CHT-S (−1.7% ± 0.07% growth per generation).

Plotting the fitness costs against the corresponding RSA survival values for each mutant line revealed no clear correlation between these two parameters in the CHT isolates (Spearman *r* = 0.14, ns) (Fig. 4C). Notably, this analysis reveals that R561H is both fit and exhibits high resistance (Fig. 4C).

### K13 R561H edited lines recover fastest post-DHA treatment

**Fig 5:**
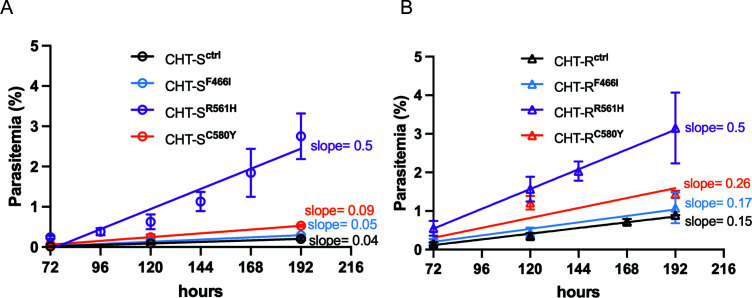
Post-DHA treatment recovery assays in the edited lines show the differential impact of different mutations on recovery. Linear regression analysis of parasite growth, measured as percent parasitemia from 72 to 192 h post-treatment following a 6-h exposure to 700 nM DHA during the 0–3 h ring stage, was performed for *k13*-edited lines in the CHT-S (**A**) and CHT-R (**B**) genetic backgrounds. In CHT-S, the R561H mutation exhibited the steepest recovery trajectory (slope = 0.50, *R*^2^ = 0.8), indicating significant post-treatment growth, whereas F446I and C580Y showed minimal recovery (slopes = 0.09 and 0.05; *R*^2^ = 0.90 for both), comparable to the control line (slope = 0.04, *R*^2^ = 0.9). In CHT-R, R561H again demonstrated the highest recovery (slope = 0.51, *R*^2^ = 0.89), followed by C580Y (slope = 0.26, *R*^2^ = 0.85) and F446I (slope = 0.17, *R*^2^ = 0.84), with the control line showing a slope of 0.15 and *R*^2^ = 0.94. Data represent means ± SEM from two to four biological replicates, each performed in duplicate (Files S6 and S7).

Following a 6 h treatment of 0–3 h ring-stage parasites with 700 nM DHA, growth recovery was monitored by measuring parasitemia over 8 days (192 h) (Fig. 5). Linear regression analysis revealed distinct recovery patterns among edited lines. For CHT-S isolates (Fig. 5A; File S6), the R561H mutation displayed the steepest recovery trajectory with a slope of 0.5 and an *R*^²^ value of 0.8, indicating significant post-DHA treatment growth. This was notably higher compared to the other mutations: F446I and C580Y showed minimal growth recovery with slopes of 0.09 and 0.04, respectively, and an *R*^²^ of 0.9, comparable to the transfected control line (slope = 0.04, *R*^²^ = 0.9), suggesting that CHT-S with K13 F446I and C580Y confer negligible to minimal growth advantage in the presence of DHA. In the CHT-R background (Fig. 5B; File S6), the R561H mutation again demonstrated the highest recovery rate (slope = 0.5, *R*^²^ = 0.89), with no difference between the CHT-S and CHT-R backgrounds. The C580Y mutation displayed a moderate recovery slope of 0.26 (*R*^²^ = 0.85), while F446I had a similar recovery slope of 0.17 (*R*^²^ = 0.84), compared to the control (slope = 0.15, *R*^²^ = 0.94).

## DISCUSSION

In line with the National Strategic Plan for Malaria Elimination (2021–2025), Bangladesh’s ongoing efforts are now intensifying toward eradicating the residual pockets of malaria transmission by 2030, particularly in the southeastern remote, densely forested CHTs persisting as stable malaria hotspots bordering Myanmar and India. A critical component of this strategy is enhancing surveillance and intervention measures to prevent the establishment and spread of artemisinin-resistant *Plasmodium falciparum* strains, ensuring the sustainability of elimination efforts (4, 76). CHTs are vulnerable to the cross-border introduction of drug-resistant malaria strains due to high population mobility through the movement of refugees and migrants (5, 10, 77). ART-R associated *k13* mutations have not yet been reported from the CHTs. We and others have found K13 substitutions from Bangladesh at a very low frequency (55, 57) and through our meta-analysis of the MalariaGEN Pf7 data set (2008–2017) (Table S2), we found that these variants are not ART-R candidate or validated markers. There is a high likelihood of ART-R causing K13 substitutions emerging and spreading, considering (i) the historical dissemination of drug-resistant mutations to previous chloroquine and sulfadoxine-pyrimethamine in this region (42), (ii) independent emergence and expansion of ART-selected K13 substitutions in countries with a similar span of ACT use (39, 54, 59–61) and similar transmission settings (9, 63–65). Therefore, this study fills an important gap in knowledge and prepares the community to act if the most prevalent K13 substitutions spread or emerge and expand.

Initially, ART-R was expected to emerge slowly in the GMS, if at all, due to the combination of an artemisinin derivative with a partner drug. However, this hope waned with its first detection on the Thai-Cambodian border in 2008 (13, 78), which, over the two decades, has spread widely in Southeast Asia (35, 67) (also in Fig. 1 ; Fig. S1).

Our meta-analysis (1997–2022) identified C580Y (5.65%) as the most prevalent K13 substitution globally, followed by F446I (1.63%) and R561H (0.86%) (Table S1). These findings align with a recent broader meta-analysis by Balmer et al. (79), covering a longer period (1980–2023) and a larger data set (>100,000 *P*. *falciparum* genomes), which similarly reported C580Y, F446I, and R561H among the top global K13 substitutions.

By 2007, different validated K13 substitutions were already prevalent, before increasing in Thailand, Cambodia, and Myanmar between 2007 and 2016 (67) (Fig. 1B; Fig. S1). A strong selection pressure drove the KEL1/PLA1 co-lineage, harboring the K13 C580Y substitution, to dominance across the eastern Greater Mekong Subregion with migration eastward to Vietnam and separately westward to Thailand and Laos (36, 37, 39, 80, 81). Over the next few years, C580Y reached near fixation in the eastern GMS (Fig. 1B), with K13 C580Y mutants displaying the classical clinical ART-R phenotypes of delayed clearance (26, 78, 82) or 3-day parasite positivity (35) and the *in vitro* ART-R phenotype of increased RSA survival (23, 26, 83). Meanwhile, in Myanmar and on the Thai-Myanmar border, C580Y emerged and spread independently on a different parasite genetic background (37), with evidence of clinical and *in vitro* ART-R phenotypes (84). This is an example of a K13 substitution spreading in neighboring countries through both independent emergence and migration (37, 39, 80, 81). A similar scenario could occur in the CHTs. C580Y also emerged independently in Papua New Guinea (59) and Guyana, associated with *in vitro* ART-R (54, 61), at lower frequencies. F446I is the predominant K13 substitution in northern Myanmar and the China-Myanmar border region (Fig. 1) (62, 85). However, its association with delayed parasite clearance remains unclear, with studies reporting conflicting evidence: no clear clinical ART-R association (78), mild delayed parasite clearance (82, 86), or clear *in vivo* ART-R indicators such as prolonged parasite clearance (87) and day 3 parasitemia (85). Its association with *in vitro* ART-R RSA phenotype also remains controversial (38, 88). Similarly, R561H, which is highly prevalent in northern Thailand and North-East Myanmar (Fig. 1) and was reported at 100% prevalence at the Thai-Myanmar border in a recent 2025 study (89), has also emerged independently in Rwanda (43) (Fig. 1), where it exhibits strong clinical and *in vitro* ART-R phenotypes, consistent with observations from the GMS (82), Rwanda (43, 45, 90), and Tanzania (91).

Although validated ART-R-associated K13 substitutions have not yet been reported from Bangladesh, we detected moderate *in vitro* ART-R in isolates from the CHTs that lacked K13 substitutions (55) (Table 1). One such isolate, I-029, exhibited an *in vitro* RSA survival of 6.4% but an *in vivo* parasite clearance half-life of 2.39 h (Table 1) ( 55), which is below the WHO threshold of 5.5 h used to define clinical ART-R. We classified this isolate as *in vitro* ART-R because its *in vitro* DHA survival exceeded the 1%–2% resistance threshold. Importantly, the clinical protocol used for these Bangladeshi isolates (55) differed from prior studies that established the 5.5 h definition. In those studies (48, 78, 92), parasite clearance kinetics were measured following 3 days of artesunate monotherapy before ACT administration, allowing PC_t_₁/₂ to reflect the effect of artemisinin alone. In contrast, in our study, ACT (artemether–lumefantrine) was administered from the outset, which might accelerate parasite clearance and reduce the apparent PC_t_₁/₂, particularly when parasites remain sensitive to one of the partner drugs, thereby potentially masking partial artemisinin resistance. However, CHT-R showed a much-prolonged PC_50_ than CHT-S (Table 1). CHT-R is genomically distinct from the CHT-S, the sensitive clone (Fig. 2). Both CHT-S and CHT-R cluster closely with previous Bangladeshi (from the entire Chittagong region, including the CHTs) and Myanmar *P. falciparum* genomes, suggesting significant genetic similarity between these samples, and they belong to a shared or closely related genetic background. The extensive use of chloroquine and sulfadoxine-pyrimethamine in Bangladesh has led to pervasive resistance among *Plasmodium falciparum* strains. Our analysis from the MalariaGEN Pf7 Bangladesh data set (2008–2017) indicates that 100% of samples (*n* = 1,310) have the K76T mutation in the CRT gene (data not shown), associated with chloroquine resistance, and 99% have the S108N mutation (data not shown) in DHFR, conferring pyrimethamine resistance, including our CHT isolates (Table 1). While CHT-S maintains a wild-type *Pfmdr1* genotype (3D7-like), CHT-R possesses the N86Y allele mutation, which could be associated with increased sensitivity to lumefantrine (the partner drug used in the patient’s ACT treatment) and mefloquine (93) (Table 2).

Through gene editing followed by RSA phenotyping analyses, we provide definitive evidence that the K13 R561H and C580Y substitutions can confer *in vitro* ART-R in both CHT backgrounds. K13-R561H substitution shows the highest level of resistance in both the CHT backgrounds (Fig. 3A). In the CHT-S strain, the level of resistance is comparable to the highest levels of R561H-induced resistance reported in other Cambodian and Thai isogenic backgrounds, such as Cam3.II (13.2%), Thai 2 (14.2%), and Thai 5 (12.7%) (31) (Fig. 3B). However, the extreme resistance phenotype observed in the CHT-R background, with 30% survival mediated by the R561H mutation (Fig. 3A), has not been previously reported in any *k13*-engineered line. Even though the fold increase of resistance mediated by R561H is lower than in the sensitive strain (4.8- vs 12-fold), the final resistance level is much higher, indicating that the CHT-R background can support high-level resistance, with RSA values plateauing at extreme levels. This suggests that R561H enhances pre-existing resistance mechanisms in the CHT-R background, but its contribution reaches a saturation point rather than producing an additive or multiplicative effect. C580Y also generated a higher resistance level in the CHT-R background and exhibited mean survival rates of 12.2% in CHT-R, compared to 4.2% in CHT-S (Fig. 3A). These resistance levels align with resistance levels observed in Southeast Asian (4.7%–9.24% survival, Fig. 3B) and African backgrounds (4.8%–15.5% survival, Fig. 3B), with the exception of Tanzanian (F32) and Chinese (FCC1/HN) isolates, where the introduction of C580Y did not result in ART-R (Fig. 3B), suggesting the importance of a genetic background for a C580Y mutation to cause resistance. In contrast, F446I did not alter susceptibility in the sensitive CHT background (Fig. 3A), consistent with previous findings from F446I editing in Dd2, 3D7, and FCC1/HN (Fig. 3B). In the resistant background, the RSA increase was by ~1.2-fold (Fig. 3A). The only instance where F446I was associated with resistance was reported by Siddiqui et al. (32), where editing the mutant allele back to wild type reversed resistance, reducing ART susceptibility by twofold in a Myanmar isolate (F09A32, Fig. 3B). Our findings from R561H and C580Y editing and phenotyping emphasize that Bangladesh’s genetic backgrounds could support high resistance if these key mutations emerge or are spread from the GMS.

We posited that beyond the varied resistance levels imparted by distinct *k13* alleles, the fitness costs associated with these mutations might also be crucial in determining the viability and spread of these mutations in the CHTs. Head-to-head growth assays (as a proxy for asexual fitness) indicate that the three K13 substitutions generally impose minimal to no fitness costs in the CHT isolates, with one exception: F446I showed a measurable growth disadvantage in the CHT-R background despite a small per generation cost (4%) (Fig. 4), aligning with observations in 3D7 (32, 94) and Dd2 (31). C580Y was fitness neutral in both CHT-S and CHT-R backgrounds (~1% fitness cost per generation) (Fig. 4B), consistent with reports from Cambodian strains (95, 96), although C580Y has incurred fitness costs in Ugandan strains (31), suggesting that K13 C580Y may not easily spread. Notably, R561H—associated with the highest RSA survival in this study—was fitness neutral in CHT-S but advantageous in CHT-R (reached ~94% frequency at the 40th day in growth competition with a fitness gain of 5.1% per generation) (Fig. 4B). This aligns with its reported competitive advantage over C580Y in a Thai-Myanmar isolate (97) and despite documented fitness costs in a Tanzanian lab strain (F32) (31), where compensatory mutations may underlie its recent expansion. Collectively, these results indicate that CHT genetic backgrounds can support high-resistance K13 alleles without substantial fitness trade-offs, facilitating their potential persistence and spread. Taken together, these findings show that the CHT genetic backgrounds can support resistant *k13* alleles such as R561H and C580Y with minimal fitness constraints, whereas F446I exhibits a clear background-specific disadvantage.

Growth of these *k13*-engineered lines following 700 nM DHA treatment (the pharmacologically relevant concentration for treatment) might be another proxy for determining the resilience of spread under drug pressure in the population. R561H consistently exhibited the highest recovery rates in both backgrounds, followed by C580Y, with F446I showing limited growth improvement, post-DHA treatment (Fig. 5). These results suggest that the R561H mutation that confers a greater DHA resistance also confers a robust and consistent growth advantage after DHA exposure, independent of genetic background. In contrast, the C580Y mutation exhibits a background-dependent effect, with improved recovery only in the CHT-R setting. F446I, in contrast, showed minimal growth recovery across both backgrounds, consistent with its limited resistance profile.

Taken together, our *in vitro* results indicate that recent isolates from the CHTs can develop and maintain high-level ART-R when acquiring prevalent K13 substitutions like R561H and C580Y. Moreover, K13-independent ART-R backgrounds have mechanisms to sustain higher levels of resistance mediated by K13-R561H and C580Y without fitness trade-offs for these mutations. This scenario suggests that if such parasitic genetic backgrounds acquire such K13 mutations in the CHTs, they could lead to more pronounced delayed clearance in patients, robust growth of the parasites, and potential spread, contingent on the transmission dynamics within the population. In endemic regions, the spread of artemisinin-resistant parasites depends not only on their ability to withstand drug pressure and sustain asexual growth but also on their mosquito-stage transmission competence. Transmission fitness, the capacity to infect mosquitoes, complete the mosquito stage, and successfully reinfect humans, acts together with resistance level and asexual fitness to determine whether resistant genotypes can establish and spread in a population. While our *in vitro* data demonstrate that certain K13 mutations (R561H and C580Y) confer high-level resistance in the CHT isolates and carry minimal asexual fitness costs, we did not assess transmission-stage fitness in this study. This omission represents a key limitation, as K13 mutations may incur stage-specific fitness costs or gains that influence their epidemiological trajectory. Notably, no recent K13 or genomic surveillance data have been reported from Bangladesh since 2019, leaving it unclear whether K13-mediated ART-R has emerged or spread in the region. Given the high-risk factors of cross-border migration and environmental similarities to the GMS, it is imperative to intensify molecular surveillance efforts in the CHTs to detect such an emergence early and prevent the establishment and spread of ART-R.

## MATERIALS AND METHODS

### Data collection and analysis for K13 substitutions

WWARN Artemisinin Molecular Surveyor K13 data (66) was accessed in October 2023. In total, we included data from 242 WWARN publications covering 72,375 samples. To ensure our database was as comprehensive as possible, we performed an additional literature search for relevant, recent papers published after 2020 to supplement the WWARN database. We searched the Web of Science and PubMed with the following string: “(K13 OR kelch OR kelch13 OR pfkelch13) AND (half-life OR parasite clearance OR resistance),” restricting results to articles only. We also searched “Kelch13 mutations falciparum artemisinin” in Google Scholar for articles published between 2022 and 2023. This search added 10 publications (47, 51, 67, 70, 98–103). We excluded articles reporting K13 double substitutions without genotyping data.

### Data limitations

Data collection in GMS declined in the last 5 years as transmission went down with additional focus in Africa; hence, many SEA regions do not have data in recent years. In our analysis, we did not demarcate the sample proportion of Africa and South-East Asia.

### Sample collection

Patient isolates CHT-S (I-001) and CHT-R (I-029) were collected from *P. falciparum*-infected patients from the Bandarban district in the CHTs in 2018 and 2019.

### *Plasmodium falciparum* culture

*P. falciparum* asexual parasites were cultured at 4% hematocrit with O+ human red blood cells (Biochemed Services, Winchester, VA, USA, and Interstate Blood Bank, Memphis, TN, USA) suspended in complete medium containing RPMI 1640 with l-glutamine (Gibco, Life Technologies), 50 mg/L hypoxanthine (Calbiochem, Sigma-Aldrich), 25 mM HEPES (Corning, VWR), 2 g/L D-glucose (Sigma), and 20 mg/L gentamicin (Gibco, Life Technologies), supplemented with 0.5% AlbuMAX II and 0.22% NaHCO_3_ in 5% CO_2_, 5% O_2_, and 95% N_2_ gas mixture (104). Patient isolate CHT-R (I-029) was culture-adapted using the above media at 10% hematocrit.

### DNA extraction for whole-genome sequencing

Adapted *P. falciparum* cultures were expanded to 5% parasitemia with mature stages. RBCs were lysed with 0.05% (wt/vol) saponin, washed with 1× PBS, and genomic DNA was isolated after RNase treatment from the parasite pellets. Genomic DNA was extracted using the QIAamp DNA Blood Mini Kit (Qiagen). The quantity and quality of gDNA were quantified by Nanodrop and Tapestation using the gDNA tape. One to five micrograms of gDNA was used for library preparation.

### Whole-genome sequencing and variant calling

Whole genome libraries of the isolates were prepared and sequenced across one lane (along with 13 other samples not reported in this manuscript) of an Illumina NextSeq 2000 P2 (300 cycles) flow cell at the Genomics and Bioinformatics Core Facility, Notre Dame (https://genomics.nd.edu). Libraries were prepared using the NEBNext Ultra II DNA Library Prep kit with Covaris sonication. Libraries were sonicated to an average size of 350 bp. Libraries were quality assessed using a combination of KAPA HiFi qPCR, TapeStation DNA HS Assay, and Qubit HS DNA assays. Libraries were pooled in equimolar amounts for sequencing on a NextSeq 2000 P2 (300 cycles) flow cell using paired 150 bp reads. Secondary analysis was done using Illumina’s onboard DRAGEN software for BCL to Fastq conversion.

### Read alignment and variant calling

We followed the analysis from MalariaGEN Pf7 bioinformatics methods (72). The fastq files were trimmed, and adaptors were removed using trimmomatic. The QC-controlled reads were aligned to *Plasmodium falciparum* 3D7 reference genome using bwa mem with default parameters. The sam files were indexed and converted to bam files using samtools. The GATK pipeline was used to remove PCR duplicates and recalibrate base quality scores. GATK-HaplotypeCaller was used for variant calling using default parameters except the –sample_ploidy 1 parameter. All hypervariable, centromeric, and subtelomeric regions were discarded, and only the 21 MB core genome was considered for downstream analysis. Joint genotyping was performed using the –GenotypeVCF command. Hard filtering was used to remove low-quality variants (DP < 20). SNPs and INDELs were separated with the GATK --selectVariant command. SNPs and INDELs were separated. SNPs were annotated using SnpEff. SNPs in candidate genes were evaluated. Only high-quality biallelic SNPs in the core genome were used for downstream analysis.

### Principal component analysis

Principal component analysis was performed on genomes from Myanmar, Bandarban, and Ramu in CHTs, Bangladesh (MalariaGEN Pf7), and our CHT samples. For population structure and PCA analyses beyond *k13*, we used the MalariaGEN Pf7 data set, because it provides whole-genome data, unlike WWARN. Biallelic core SNPs from Pf7 genomic data of selected samples (metadata are included in Source Table S2) were extracted by bcftools using Plink after linkage pruning with a 10 bp window step size for a 50 kb window with a linkage threshold (*r*^2^) < 0.1. The percent variance was explained by converting the ratio of eigenvalues to the sum of the eigenvalues to percentage. Principal component analysis was done using Python 3.0 (details in “Data availability”) on 696 samples from seven countries: Thailand (*n*_total_ = 33), Cambodia (*n*_total_ = 154), Bangladesh (*n*_total_ = 93), Vietnam (*n*_total_ = 95), Myanmar (*n*_total_ = 198), India (*n*_total_ = 100), and Indonesia (*n*_total_ = 21), as well as newly isolated CHT-S (I-001) and CHT-R (I-029). Yearwise breakdown of samples from each country: Bangladesh (*n*_₂₀₀₈_ = 13; *n*_₂₀₀₉_ = 15; *n*_₂₀₁₂_ = 49; *n*_₂₀₁₆_ = 4; *n*_₂₀₁₇_ = 12); Cambodia (*n*_₁₉₉₃_ = 5; *n*_₂₀₀₈_ = 1; *n*_₂₀₀₉_ = 2; *n*_₂₀₁₂_ = 18; *n*_₂₀₁₃_ = 2; *n*_₂₀₁₆_ = 60; *n*_₂₀₁₇_ = 66); India (*n*_₂₀₁₆_ = 30; *n*_₂₀₁₇_ = 64; *n*_₂₀₁₈_ = 6); Indonesia (*n*_₂₀₁₅_ = 7; *n*_₂₀₁₇_ = 14); Myanmar (*n*_₂₀₁₁_ = 9; *n*_₂₀₁₂_ = 33; *n*_₂₀₁₃_ = 11; *n*_₂₀₁₄_ = 29; *n*_₂₀₁₅_ = 14; *n*_₂₀₁₆_ = 46; *n*_₂₀₁₇_ = 56); Thailand (*n*_₂₀₀₁_ = 2; *n*_₂₀₀₂_ = 2; *n*_₂₀₀₃_ = 1; *n*_₂₀₀₄_ = 1; *n*_₂₀₀₅_ = 4; *n*_₂₀₀₇_ = 1; *n*_₂₀₁₁_ = 8; *n*_₂₀₁₂_ = 3; *n*_₂₀₁₇_ = 11); and Vietnam (*n*_₂₀₀₀_ = 1; *n*_₂₀₀₉_ = 9; *n*_₂₀₁₀_ = 1; *n*_₂₀₁₂_ = 10; *n*_₂₀₁₅_ = 8; *n*_₂₀₁₆_ = 5; *n*_₂₀₁₇_ = 55; *n*_₂₀₁₈_ = 6).

### CRISPR/Cas9 editing of *k13* mutations

Editing of the *k13* was performed using the pDC2-coSpCas9-k13guide-gRNA-h*dhfr* all-in-one plasmid that contains a *P. falciparum* codon-optimized Cas9 sequence, a human dihydrofolate reductase (h*dhfr*) gene expression cassette (conferring resistance to WR99210), and donor template (containing the respective nonsynonymous and shield/binding site mutations or only shield/binding site mutations) (plasmid generously provided by Dr. David Fidock) (31, 105). A volume of 80 µL of packed RBCs containing 5%–8% ring parasitemia was electroporated with 50 µg of the purified plasmid resuspended in Cytomix using a BioRad Gene Pulser Xcell Electroporation System. Transfected parasites were maintained under 5 nM WR99210 (Jacobus Pharmaceuticals) to select for edited parasites till 6 days post-transfection, after which the drug was removed from the media. Parasite cultures were monitored for recrudescence microscopically for up to 6 weeks post-electroporation.

### Validation of *k13* edits—PCR and sequencing

*k13* edit was validated by both direct PCR using Phusion Blood Direct PCR Kit (ThermoFisher Inc.) and from genomic DNA isolated from the bulk transfected cultures and their clones. For PCR, 2 µL culture/25 ng genomic DNA was added as the template in a 20 µL reaction. PCR product was purified using the QIAquick PCR Purification Kit (Qiagen) and Sanger sequenced. The primers used for amplification and Sanger sequencing are k13-1270-Fw: GAAAGTGAAGCCTTGTTGAAAGAAGCAG and k13-UTR-rev: aaatgtgcatgaaaataaatattaaagaag.

### Cloning of parasites by limiting dilution

Transfected *k13*-edited bulk culture or the CHT isolate (I-029) was diluted, and low-density seeding was performed on a 96-well plate at 0.25 parasites per well as previously described (106). Plates were maintained in a gassed incubator for a month with media changes and screening every week for parasite-positive wells. Screening was performed by qPCR using the Phusion Blood Direct PCR kit (ThermoFisher Inc.), supplemented with 1× SYBR, beginning in week 2 and continuing in weeks 3, 4, and 6. Two microliters of culture was used in a 10 μL reaction and amplified using forward and reverse primers of the *pfcrt* gene. PCR amplification was measured using the ABI 7900HT, with a 5 min denaturation at 98°C, followed by 40 cycles of 95°C for 1 s, 53°C for 5 s, and 65°C for 15 s, then final extension at 65°C for 60 s. Primers used for amplification are CRT-F: GGGTGATGTTGTAAGAGAACCA and CRT-R: ACGAACAAGCCATTTGATATTA. Positive wells with a CT score of 25 or lower were transferred to 1 mL wells at 5% Hct in 24-well plates for microsatellite analysis, expansion, cryopreservation, and characterization.

### *In vitro* 72-h drug susceptibility assa**y**

Synchronized 0–12 h rings were grown for 72 h in the presence of different concentrations of drugs in 96-well plates at 1% hematocrit and 0.5% starting parasitemia. Growth at 72 h was measured by SYBR Gold (Invitrogen) staining of parasite DNA on a Plate Reader. A 2-point dilution series of the drugs was carried out in duplicate with three to five biological replicates. Relative fluorescence units were measured at an excitation of 494 nm and emission of 530 nm on a Plate Reader and analyzed using GraphPad Prism version 8 (GraphPad Software, La Jolla, CA, USA). IC_50_ values were determined with the curve-fitting algorithm log(inhibitor) versus response–Variable slope.

### Ring-stage survival assay

The RSA_0–3h_ was performed as previously described (23, 29). Late-stage segmented schizonts, purified with Percoll, were briefly cultured with fresh red blood cells for 3 h, followed by sorbitol synchronization. Cultures containing 0–3 h rings were adjusted to a 2% hematocrit and 1% parasitemia and seeded into a 24-well plate with 2 mL of complete medium per well, containing either dihydroartemisinin at 700 nM or 0.1% dimethyl sulfoxide (DMSO) as a control. After 6 h at 37°C, the cultures were washed and transferred to drug-free medium. Subsequently, after 66 h (equivalent to 72 h from initial seeding), thin blood smears were prepared, and survival rates were microscopically assessed by counting the proportion of viable rings with normal morphology after staining with Giemsa. Parasitemia was estimated based on counts from 10,000 red blood cells in controls and 20,000 RBCs in treated samples. Survival percentage rates were determined by multiplying the ratios of viable parasitemia in DHA-exposed parasites to DMSO-treated controls by 100 and expressed as a percentage, with a survival rate ≥1% indicating the threshold for resistance (29).

### Post-DHA-treated growth assay

Cultures containing 0–3 h ring-stage parasites were diluted to a starting parasitemia of 1% and treated with 700 nM DHA for 6 h. After 6 h, DHA was washed off, and parasites were returned to drug-free media. Parasitemia was measured by microscopy beginning at 72 h post-treatment and continued at designated time points up to 192 h (8 days). Media changes were performed at 72, 120, and 168 h following DHA treatment.

### Pairwise head-to-head competitive growth assay

We conducted head-to-head pairwise competition growth assays between each *k13*-edited line and their corresponding isogenic control transfected line (*k13* shield mutations). We performed these co-culture assays in 96-well plates by mixing the lines at a 1:1 ratio of synchronized rings, each strain at 0.5% with a total of 2% hematocrit in 200 µL culture/well and measuring the proportion of one strain to another following established protocols (73, 107). Synchronized ring-stage parasites were used to initiate the assay (day 0). Cultures were maintained in a gassed incubator with media changes or splitting every other day for 40 days. A portion of each culture was collected at days 0, 7, 14, 30, and 40, which were stored at −20°C for genotyping. The removed volume was replaced with fresh complete media and RBCs to maintain a consistent 200 μL volume. They were performed in three biological replicates with three technical replicates each in a co-culture competition experiment. The *k13* gene from chromosome 13 on the genome was amplified using the Phusion RBC PCR kit using K13-1270-fw: GAAAGTGAAGCCTTGTTGAAAGAAGCAG and K13-UTR-rev: aaatgtgcatgaaaataaatattaaagaag primers. PCR products were quantified and diluted to 5 ng/μL, and 2 μL (10 ng) was used per reaction. Growth of the lines with different *k13* SNPs in the same background was measured by qPCR using SNP genotyping RhAmp SNP kit (IDT) (108), with primers targeting the *k13* edited region, in a 10 μL reaction volume, including 5.3 μL of combined Genotyping Master Mix and Reporter Mix (20:1 ratio), 0.5 μL of 20× SNP Assay, 2.2 μL of water, and 2 μL of PCR product. qPCR was run on an ABI system using the SNP genotyping protocol. Allele-specific primers (see below) were designed using IDT’s rhAMP genotyping tool and include universal primer binding tails (GT1, GT2, GT3, and GT4) required for amplification with rhAMP Master Mix. Mutant alleles were detected via the VIC channel and wild-type alleles via FAM. For each sample, ΔCt was defined as Ct(VIC) − Ct(FAM). We computed the mutant-to-wild-type ratio as fold change = 2^ΔCt^, and the percentage of mutant allele as % mutant = [fold change/(fold change + 1)] × 100. Samples collected on days 0, 10, 14, 30, and 40 were analyzed to quantify allele frequencies over time.

### Fitness cost determination at competition assay endpoint

Fitness cost per generation for each K13 mutant line was estimated relative to its isogenic wild-type control using the equation: P′ = *P* × (1 − *x*)^*n*^, where *P* represents the initial parasitemia, *P*′ is the parasitemia measured at the assay endpoint, *n* is the number of parasite generations, and *x* is the fitness cost per generation (31). We performed our fitness calculations for 30 days, corresponding to 15 parasite generations. Input data for this calculation were derived from the results of pairwise competition assays between each edited line and its respective control, as described above. rhAmp primers used are as follows: K13-F446I, Allele Primer 1, rhAmp-F/ACCATCAAATCCACCTATACAAAAArUACTA/GT4/; Allele Primer 2, rhAmp-Y/ACCATCAAATCCACCTATACAAAATrUACTA/GT4/; and Locus primer, GCACCCATACCAAAAGAGATTTAAGTGAAArGTGAA/GT2/; K13-R561H, Allele Primer 1, rhAmp-F/ACCTCTACCCCATGCTTTCTATACrGATGA/GT1/; Allele Primer 2, rhAmp-Y/CACCTCTACCCCATGCTTTCTATATrGATGA/GT1/; and Locus primer, GCTGTGGTGTTACGTCAAATGGTTrAGAAT/GT1/; K13-C580Y, Allele Primer 1, rhAmp-F/GCCAAGATCATCAGCTATGTGrUGTTG/GT3/; Allele Primer 2, Y/CGCCAAGATCATCAGCTATGTARUGTTG/GT3/; and Locus primer, GCTCTCACCATTAGTTCCACCAArUGACA/GT1/.

## Data Availability

Codes used for principal component analysis are deposited at https://github.com/NirjharBhattacharyya/PCA_for_CRISPR_Paper. The raw whole-genome sequencing data from CHT-S and CHT-R have been deposited in the NCBI Sequence Read Archive (SRA) under accession number PRJNA1264464.

## SUPPLEMENTAL MATERIAL

File S1 mbio.03691-25-s0001.xlsx Source data for Fig. 1A.

File S2 mbio.03691-25-s0002.xlsx Source data for Fig. 1B.

File S3 mbio.03691-25-s0003.xlsx Source data for Fig. 3A.

File S4 mbio.03691-25-s0004.xlsx Source data for Fig. 3B.

File S5 mbio.03691-25-s0005.xlsx Source data for Fig. 4A.

File S6 mbio.03691-25-s0006.xlsx Source data for Fig. 5A.

File S7 mbio.03691-25-s0007.xlsx Source data for Fig. 5B.

Supplemental Figures mbio.03691-25-s0008.docx Figures S1 to S4.

Source Tables S1 and S2 mbio.03691-25-s0009.xlsx Source data tables for Fig. S1 and S2.

Supplemental Tables mbio.03691-25-s0010.xlsx Tables S1 to S3.

ASM does not own the copyrights to Supplemental Material that may be linked to, or accessed through, an article. The authors have granted ASM a non-exclusive, world-wide license to publish the Supplemental Material files. Please contact the corresponding author directly for reuse.
