# Supplementary material for: Functional assessment of prevalent kelch13 mutations reveals high-level artemisinin resistance potential in Bangladeshi Plasmodium falciparum
Source: mBio. 2026 Mar 17;17(4):e03691-25. doi: 10.1128/mbio.03691-25 (PMC13064676; doi:10.1128/mbio.03691-25)
Supplement: Supplemental Figures — Figures S1 to S4. [file mbio.03691-25-s0008.docx]

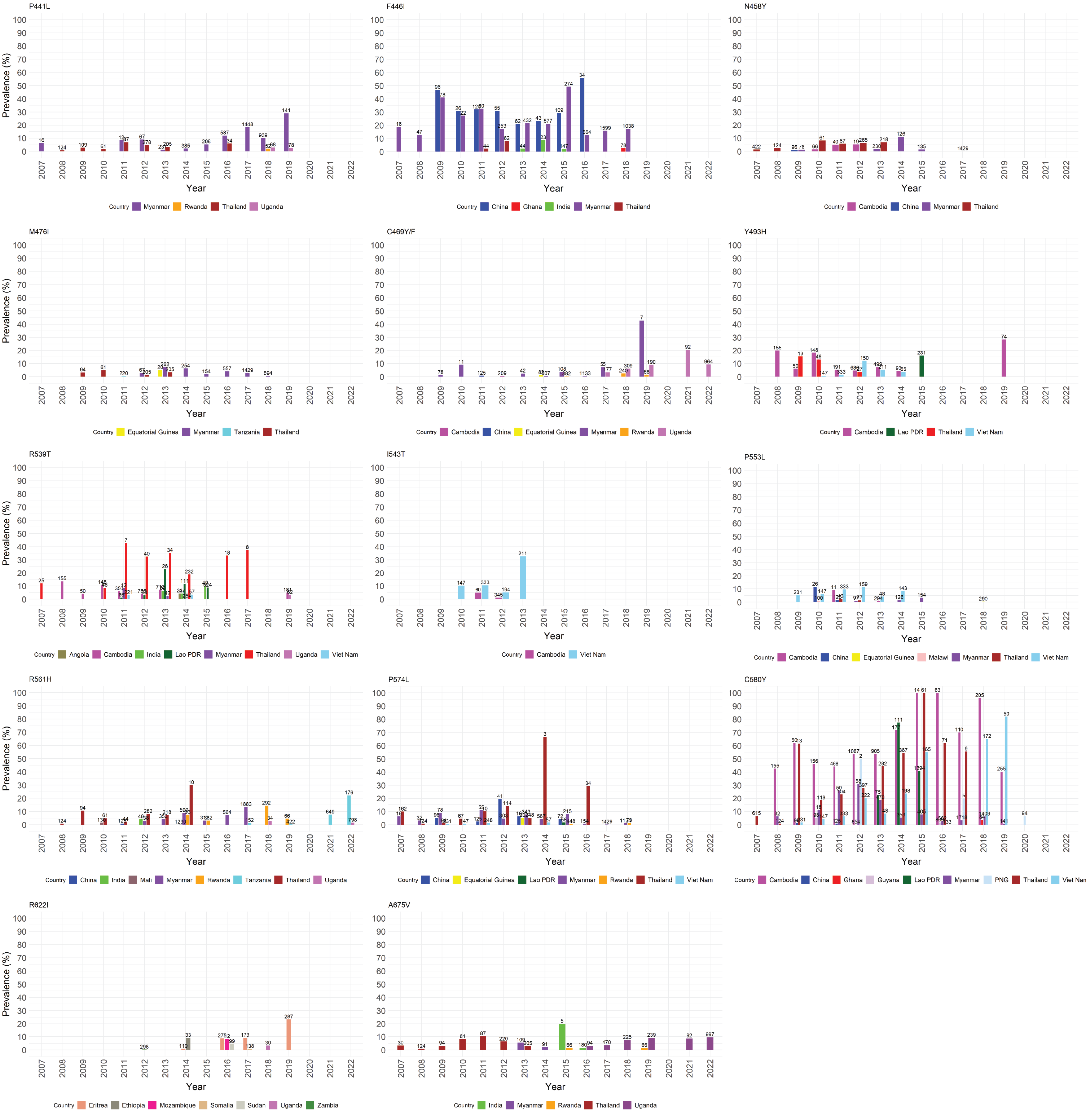


**Supplementary Figure S1**: Spatiotemporal trends in the prevalence of 14 WHO-validated K13 substitutions from 2007 to 2022. The y-axis indicates mutation-specific prevalence (%), and the x-axis shows the corresponding year. Each colored bar represents a different country, with the total number of samples analyzed shown above each bar. Data are compiled from **Source Table S1**.


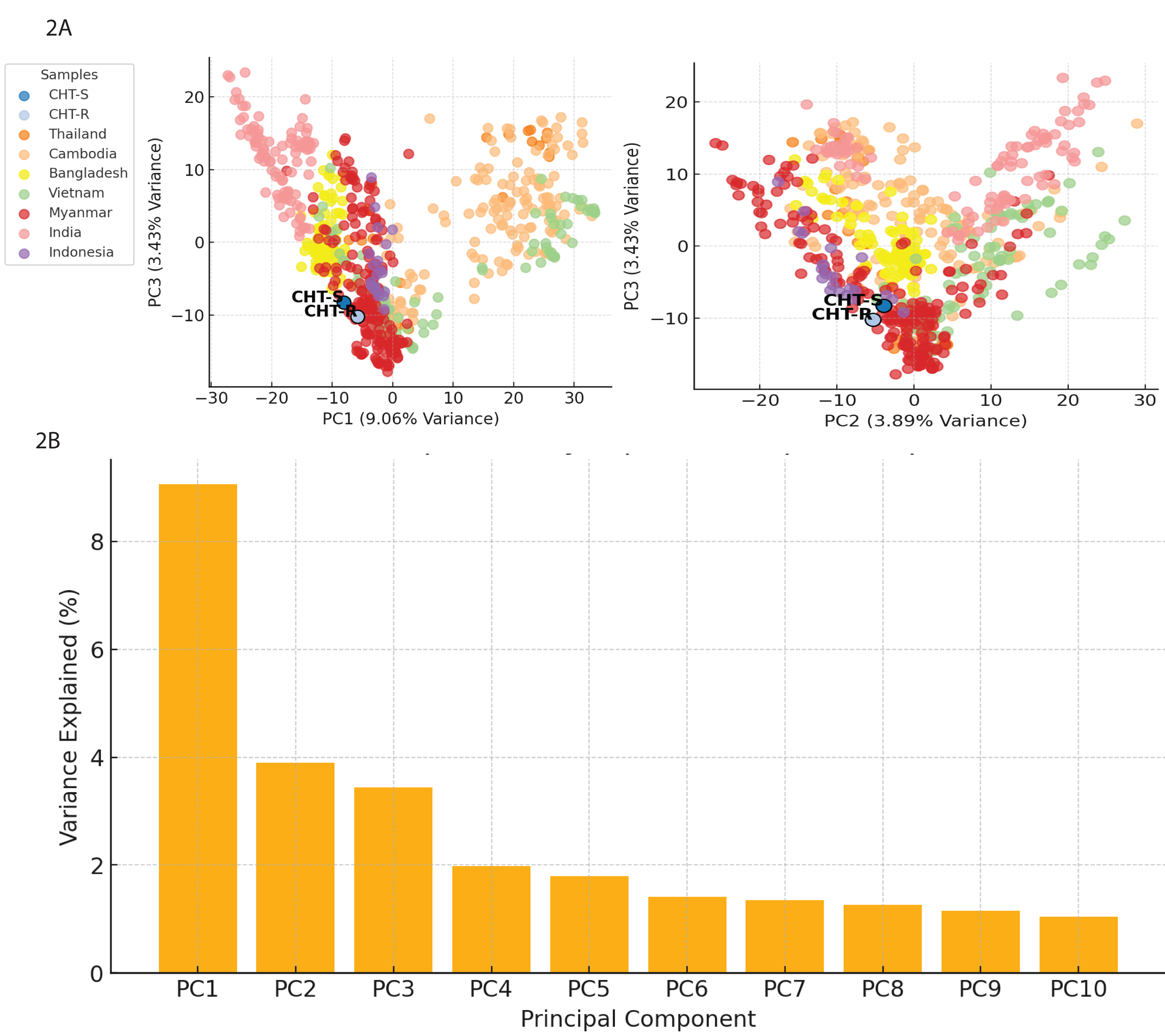


**Supplementary Figure S2. A.** Pairwise Principal Component Analysis (PCA) using Principal Component 1, 2, and 3. PC1 and PC2 explain 9.06% and 3.43% variance and PC2 and PC3 explain 3.89% and 3.43% variance of the multisample genomic data. **B.** Variance explained in percentage (%) by the first ten principal components in the PCA.


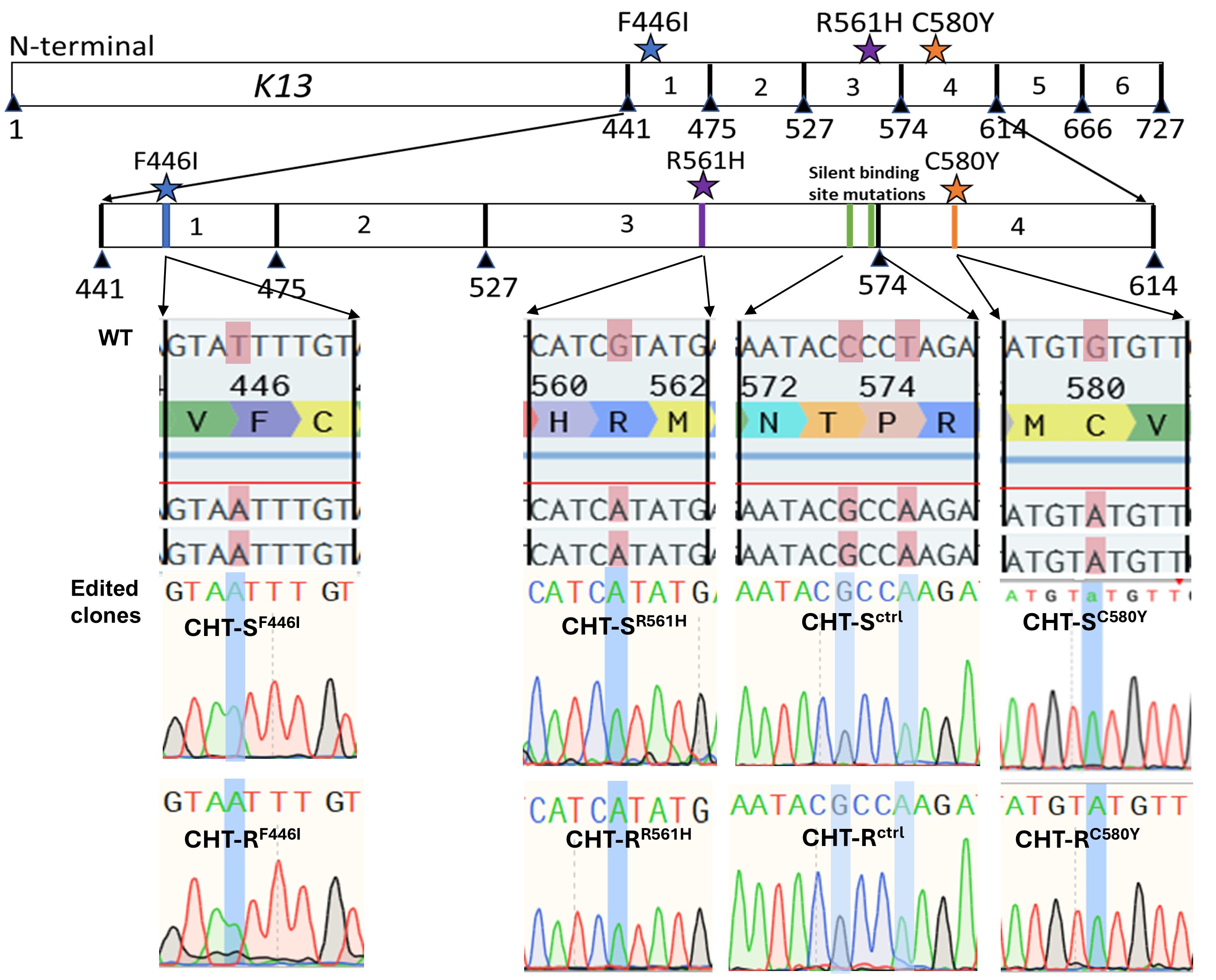


**Supplementary Figure S3.** CRISPR-Cas9 genetic editing of K13 F446I, R561H, C580Y and silent binding site control mutations (ctrl) in CHT-S and CHT-R. Cartoon shows location of the K13-propeller domain substitutions. Reference sequence of 3D7 on top and Sanger sequence and chromatogram analysis of an edited clone below. Highlighted bases show the WT and edited alleles of clones. CHT-S^ctrl^ and CHT-R^ctrl^ parasites contain only synonymous, phenotypically silent mutations.


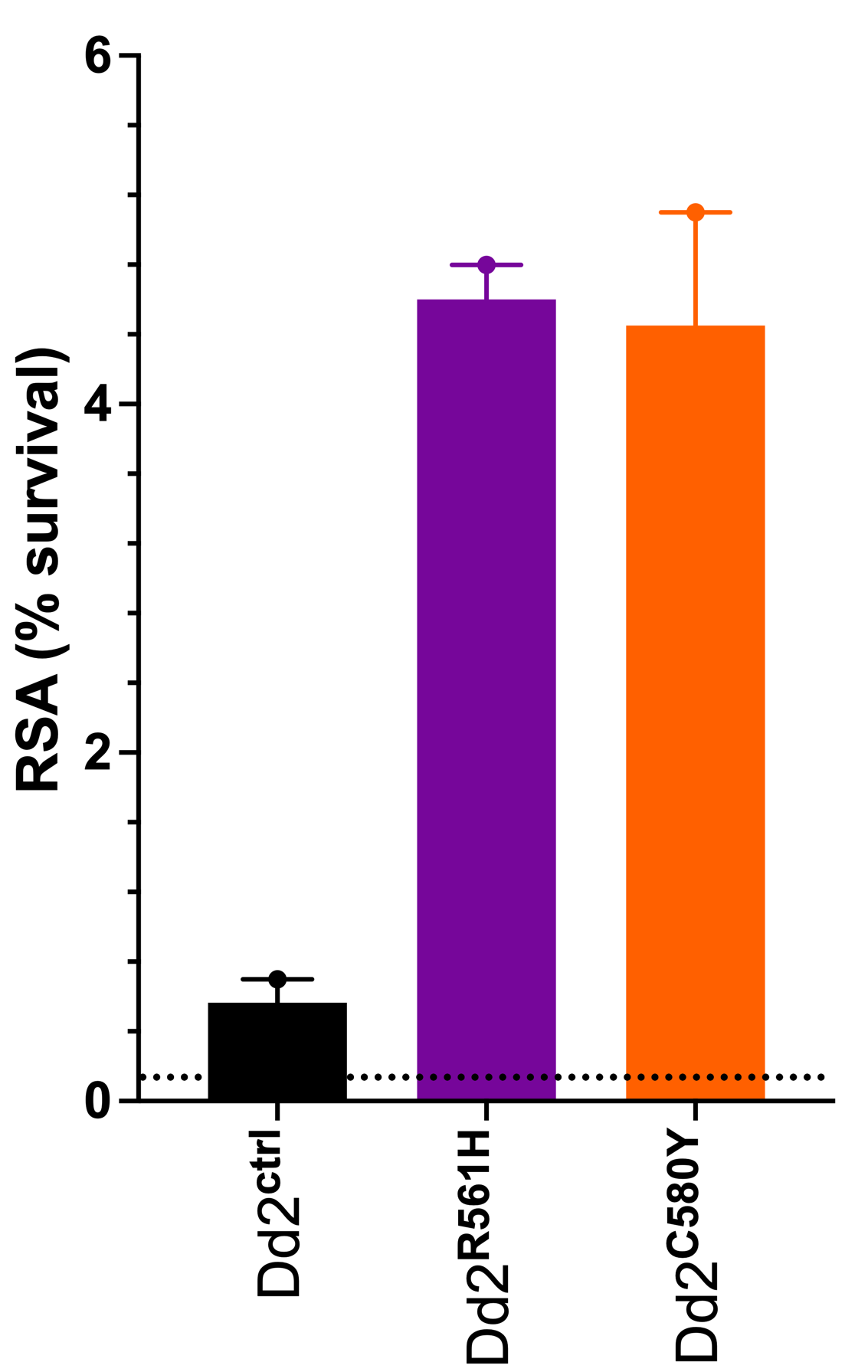


**Supplementary Figure S4.** RSA survival of silent, R561H and C580Y K13 substitutions in Dd2. Parasites were graciously donated by Dr. David Fidock.
